# How does lesion size affect the pooled effect of traction-assisted endoscopic submucosal dissection on procedure time? A meta-regression

**DOI:** 10.1186/s12957-019-1699-0

**Published:** 2019-09-10

**Authors:** Sheng-Wei Cheng, Chun-Chao Chang, Ying-Fong Su, Yi-No Kang

**Affiliations:** 1 Division of Gastroenterology, Department of Internal Medicine, Taipei Municipal Wan Fang Hospital, Taipei Medical University, Taipei, Taiwan, Republic of China; 2 Division of Gastroenterology and Hepatology, Department of Internal Medicine, Taipei Medical University Hospital, Taipei, Taiwan, Republic of China; 3 Division of Gastroenterology and Hepatology, Department of Internal Medicine, School of Medicine, College of Medicine, Taipei Medical University, Taipei, Taiwan, Republic of China; 4 School of Medicine, College of Medicine, Taipei Medical University, Taipei, Taiwan, Republic of China; 5 Evidence-Based Medicine Center, Wan Fang Hospital, Taipei Medical University, Taipei, Taiwan, Republic of China; 6 Institute of Health Policy & Management, College of Public Health, Taipei, Taiwan, Republic of China

## Abstract

The purpose of this letter was to explore how lesion size affects the pooled effect of traction-assisted endoscopic submucosal dissection on procedure time. Our meta-regression showed that lesion size negatively associated with the effect of traction-assisted endoscopic submucosal dissection on procedure time (estimate point = − 1.02; 95% confidence interval, from − 1.58 to − 0.46). We also confirmed this result in different statistical models including fixed effect regression and two mixed effects regression models.

**Electronic supplementary material:**

The online version of this article (10.1186/s12957-019-1699-0) contains supplementary material, which is available to authorized users.

Dear Editor,

We read with interest the study published in *World Journal of Surgical Oncology* which synthesized seven randomized clinical trials with 964 cases for examining the effectiveness of traction assistance on endoscopic submucosal dissection (ESD) [1]. The research found that traction-assisted ESD (TA-ESD) has two advantages including shorter procedure time and lower perforation rate, and TA-ESD reaches similar en bloc resection rate and complete resection rate to traditional ESD (T-ESD). However, there is an extremely high heterogeneity (*I*^2^ = 87%) in their pooled result of procedure time (main outcome). We completely agree with them about conducting subgroup analysis for exploring the source of the heterogeneity according to anatomy, yet their result still reflected a high heterogeneity (*I*^2^ = 60%). As we know, an important factor affecting procedure time is lesion size. Unfortunately, they did not explore how lesion size affects procedure time. Thus, we wrote this letter to improve the understanding about the role of lesion size in the effects of TA-ESD on procedure time through using appropriate data and meta-regression.

Six out of the seven trials in the previous meta-analysis reported lesion size, and mean lesion size ranged from 15.6 mm to 36.25 mm. Based on relevant data in the previous meta-analysis, we checked that the result was not seriously affected by each single trial (Additional file 1: Figure S1), and conducted meta-regression. Our meta-regression showed that lesion size negatively associated with the effect of TA-ESD on procedure time (estimate point = − 1.02; 95% confidence interval, from − 1.58 to − 0.46; Fig. 1). This result indicated that TA-ESD needs lesser time than T-ESD for a treating bigger lesion. To be specific, TA-ESD reduces about 1 min/mm lesion when it is compared with T-ESD. We confirmed this result in different statistical models including fixed effect regression and two mixed effects regression models (Additional file 1: Table S1). Moreover, no evidence detected serious small study bias in this result (Egger’s test = − 2.85, *p* = 0.30; Additional file 1: Figure S2).

**Fig. 1:**
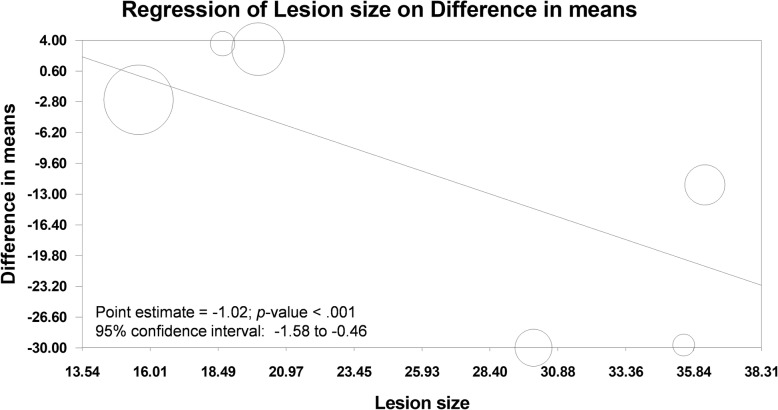
Bubble plot of meta-regression for lesion size on procedure time between traction-assisted endoscopic submucosal dissection and traditional endoscopic submucosal dissection

In summary, we successfully identified an important factor causing heterogeneity in the pooled mean difference of procedure time between TA-ESD and T-ESD. This meaningful finding fosters the understanding of the impact of lesion size on the effectiveness of TA-ESD. Based on the previous synthesis and our meta-regression, TA-ESD is worth to be considered for patients deciding to receive ESD, especially in patients with large-size lesions. Lesion size is associated with fibrosis, and fibrosis is an important factor predicting perforation after ESD [2, 3]. Nevertheless, our evidence is limited to explore the impact of TA-ESD on lesions with fibrosis because current reports presented insufficient information about baseline fibrosis. We suggest future studies to examine how traction-assisted methods influence the ESD outcomes in those having fibrosis. Furthermore, the authors presented different effects on procedure time in the stomach, colorectum, and esophagus through subgroup analysis, but they did not clarify en bloc resection rate, complete resection rate, perforation rate, and delayed bleeding rate in different lesion location. As we know, procedure view and complexity in the stomach may differ from the colorectum and esophagus [4–7]. Therefore, we anticipate further studies exploring those results with stratifications of upper gastrointestinal tract lesions and lower gastrointestinal tract lesions in the future. These further analyses will improve TA-ESD application in clinical practice.

## Additional file

Additional file 1: **Table S1.** Meta-regression of lesion size on procedure time. **Figure S1.** Forest plot of procedure time between traction-assisted endoscopic submucosal dissection and traditional endoscopic submucosal dissection. **Figure S2.** Small study effect of procedure time between traction-assisted endoscopic submucosal dissection and traditional endoscopic submucosal dissection. (PDF 63 kb)

## Data Availability

All data generated or analyzed during this study are included in this published article

## Abbreviations

ESD: Endoscopic submucosal dissection
TA-ESD: Traction-assisted endoscopic submucosal dissection
T-ESD: Traditional endoscopic submucosal dissection
