# Supplementary material for: How does lesion size affect the pooled effect of traction-assisted endoscopic submucosal dissection on procedure time? A meta-regression
Source: World J Surg Oncol. 2019 Sep 10;17:157. doi: 10.1186/s12957-019-1699-0 (PMC6737606; doi:10.1186/s12957-019-1699-0)
Supplement: Supplementary file 1 — Table S1. Meta-regression of lesion size on procedure time. Figure S1. Forest plot of procedure time between traction-assisted endoscopic submucosal dissection and traditional endoscopic submucosal dissection. Figure S2. Small study effect of procedure time between traction-assisted endoscopic submucosal dissection and traditional endoscopic submucosal dissection. (PDF 63 kb) [file 12957_2019_1699_MOESM1_ESM.pdf]

How does lesion size affect the pooled effect of traction-assisted endoscopic submucosal dissection on procedure time? A meta-regression

A letter to the editor

(Supplementary Materials)

Letter to the editor on: Xia, M., Zhou, Y., Yu, J., Chen, W., Huang, X., & Liao, J. Short-term outcomes of traction-assisted versus conventional endoscopic submucosal dissection for superficial gastrointestinal neoplasms: a systematic review and meta-analysis of randomized controlled studies. World journal of surgical oncology. 2019;17:94.

Table S1

Meta-regression of lesion size on procedure time

Fixed effect regression

	Point estimate	Standard error	Lower limit	Upper limit	Z-value	p-Value
**Slope**	-1.01940	0.28395	-1.57593	-0.46287	-3.59010	0.00033
**Intercept**	15.97709	6.69121	2.86256	29.09161	2.38777	0.01695

Mixed effects regression (method of moments)

	Point estimate	Standard error	Lower limit	Upper limit	Z-value	p-Value
**Slope**	-1.19915	0.54605	-2.26939	-0.12890	-2.19602	0.02809
**Intercept**	20.17277	14.39775	-8.04630	48.39185	1.40111	0.16118

Mixed effects regression (unrestricted maximum likelihood)

	Point estimate	Standard error	Lower limit	Upper limit	Z-value	p-Value
**Slope**	-1.15253	0.44119	-2.01724	-0.28781	-2.61233	0.00899
**Intercept**	19.09319	11.41112	-3.27219	41.45857	1.67321	0.09429

Figure S1

Forest plot of procedure time

Study name	Statistics for each study			
	**Difference in means**	**Lower limit**	**Upper limit**	***p*-Value**
Mori et al.	-57.750	-79.793	-35.707	0.000
Yamasaki et al.	-30.000	-42.680	-17.320	0.000
Ritsuno et al.	-29.700	-51.001	-8.399	0.006
Koike et al.	-12.000	-23.745	-0.255	0.045
Yoshida et al.	-2.600	-9.426	4.226	0.455
Ahn et al.	3.000	-6.007	12.007	0.514
Ban et al.	3.600	-15.523	22.723	0.712
**Total**	-16.170	-29.137	-3.204	0.015

Difference in means (95% CI)

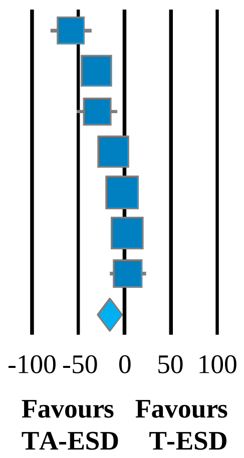

Sensitivity analysis

Study name	Statistics for each study			
	**Difference in means**	**Lower limit**	**Upper limit**	***p*-Value**
Mori et al.	-10.310	-21.148	0.527	0.062
Yamasaki et al.	-13.448	-26.678	-0.218	0.046
Ritsuno et al.	-14.321	-28.078	-0.563	0.041
Koike et al.	-17.271	-32.838	-1.705	0.030
Yoshida et al.	-19.331	-36.053	-2.609	0.023
Ahn et al.	-20.073	-35.212	-4.934	0.009
Ban et al.	-19.183	-33.463	-4.903	0.008
	-16.170	-29.137	-3.204	0.015

Difference in means (95% CI) with study removed

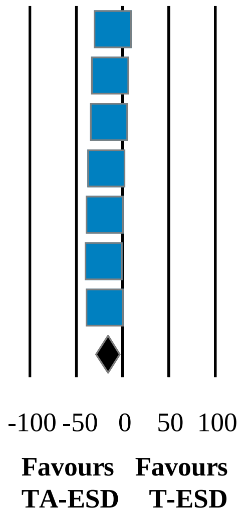

Figure S2
Small study effect of procedure time

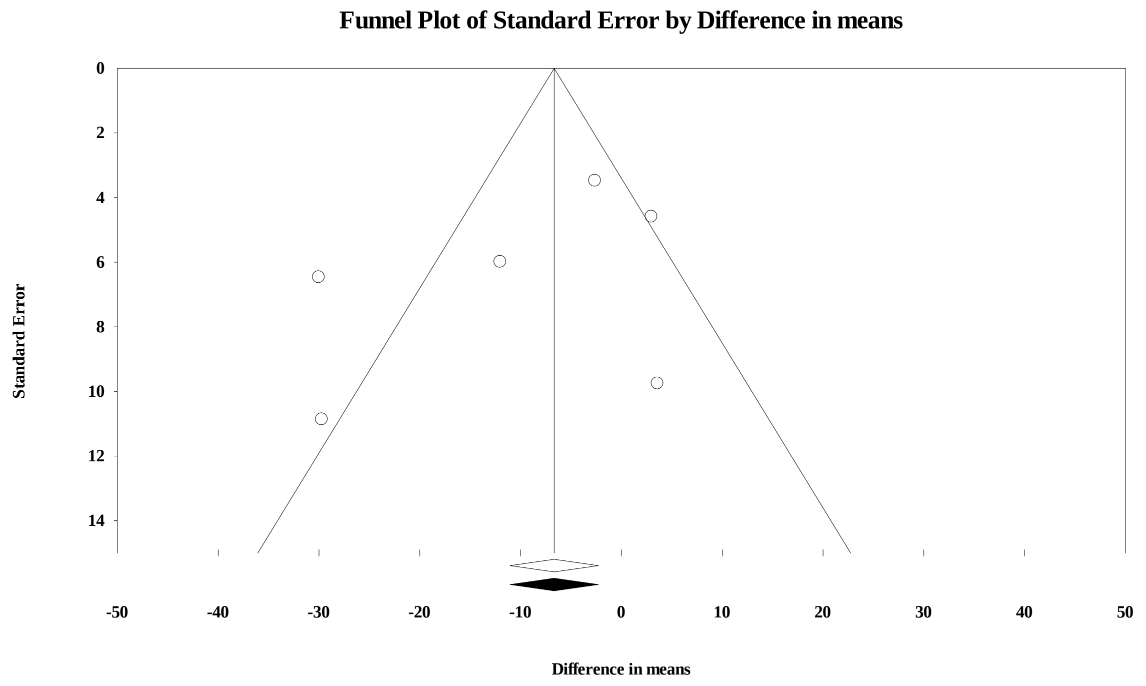

Egger's regression intercept

Intercept	-2.84985
Standard error	2.40831
95% lower limit (2-tailed)	-9.53640
95% upper limit (2-tailed)	3.83670
t-value	1.18334
df	4.00000
P-value (1-tailed)	0.15110
P-value (2-tailed)	0.30220

Begg and Mazumdar rank correlation

**Kendall's S statistic (P-Q)**	-7.00000

Kendall's tau without continuity correction

Tau	-0.46667
z-value for tau	1.31507
P-value (1-tailed)	0.09424
P-value (2-tailed)	0.18849

Kendall's tau with continuity correction

Tau	-0.40000
z-value for tau	1.12720
P-value (1-tailed)	0.12983
P-value (2-tailed)	0.25966
